# Glutathione-Binding Site of a *Bombyx mori* Theta-Class Glutathione Transferase

**DOI:** 10.1371/journal.pone.0097740

**Published:** 2014-05-21

**Authors:** M. D. Tofazzal Hossain, Naotaka Yamada, Kohji Yamamoto

**Affiliations:** Faculty of Agriculture, Kyushu University Graduate School, Fukuoka, Japan; University of Insubria, Italy

## Abstract

The glutathione transferase (GST) superfamily plays key roles in the detoxification of various xenobiotics. Here, we report the isolation and characterization of a silkworm protein belonging to a previously reported theta-class GST family. The enzyme (bmGSTT) catalyzes the reaction of glutathione with 1-chloro-2,4-dinitrobenzene, 1,2-epoxy-3-(4-nitrophenoxy)-propane, and 4-nitrophenethyl bromide. Mutagenesis of highly conserved residues in the catalytic site revealed that Glu66 and Ser67 are important for enzymatic function. These results provide insights into the catalysis of glutathione conjugation in silkworm by bmGSTT and into the metabolism of exogenous chemical agents.

## Introduction

Glutathione (GSH) conjugation is essential for the detoxification of xenobiotics [1], [2]. Several studies have also implicated conjugation reactions with endogenous compounds, such as α,β-unsaturated aldehydes and prostaglandin [2]–[4], resulting in the excretion of at least one water-soluble compound. GSH transferases (GSTs, EC 2.5.1.18) are responsible for catalysis of this conjugation and are distributed ubiquitously among aerobic organisms [5]. GSTs are cytosolic enzymes, widely distributed across both prokaryotic and eukaryotic kingdoms [6]. In mammals, there are seven GST classes (alpha, mu, pi, omega, sigma, theta, and zeta) that can be distinguished based on their primary amino acid sequence; identity is approximately 50% within a class and less that 30% between different classes [7], [8]. Six GST classes (delta, epsilon, omega, sigma, theta, and zeta) have been identified in dipteran insects, such as *Anopheles gambiae* [9] and *Drosophila melanogaster* [10], [11]. Insect GSTs can determine sensitivity to insecticides [9], [12], and since the Lepidoptera are the principal insect pests in agriculture, knowledge of lepidopteran GSTs is of great importance. We have previously characterized several GSTs in the silkworm, *Bombyx mori*, a lepidopteran model insect [13]–[19], and a sigma-class GST in the fall webworm, *Hyphantria cunea*, one of the most serious lepidopteran pests of broad-leaved trees [16]. However, there have been no reports to date on the characterization of theta-class GSTs from silkworms.

Here, we report the identification and classification of a theta-class GST isolated from *B. mori*, which we named bmGSTT. While bmGSTT shares some common substrates with human theta-class GSTs (hGSTT), it has a distinct substrate profile when compared to other *B. mori* GSTs studied to date. Furthermore, bmGSTT does not participate in the response to agents that generate oxidative stress, in contrast to previously identified *B. mori* GSTs. The activity profile of bmGSTT sheds further light on the way in which insects deal with xenobiotic agents and contributes to a more detailed understanding of the GST system in general.

## Materials and Methods

### Insects and tissue dissection

Larvae of the silkworm, *B. mori*, were reared on mulberry leaves in the Institute of Genetic Resources, Kyushu University Graduate School (Fukuoka, Japan). At day -1 fifth instar larvae, fat bodies were dissected from the larvae on ice and stored at −80°C until use. Total RNA was extracted rapidly from the dissected fat bodies with the RNeasy Plus Mini Kit (Qiagen Inc., Valencia, CA), in accordance with the manufacturer's instructions, and the resultant RNAs were subjected to RT-PCR.

### Cloning and sequencing of cDNA encoding bmGSTT

**Figure 1:**
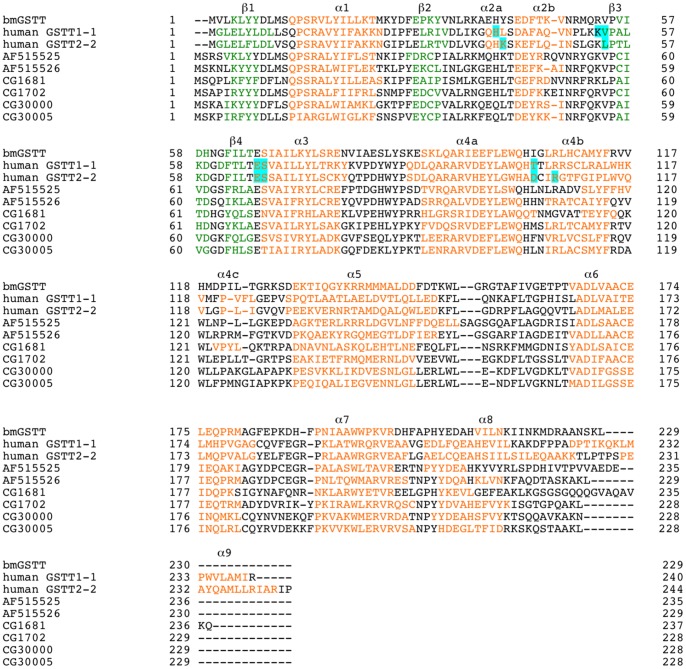
Alignment of amino acid sequences of theta-class GSTs. Sequences of GSTs were obtained in this study or from Swiss-Prot database (http://expasy.org/sprot/) and flybase (http://flybase.org): *B. mori* (present study), hGSTT1-1 (No. P30711), hGSTT2-2 (No. PCG30), *A. gambiae* (AF515525 and AF515526), and *D. melanogaster* (CG1681, CG1702, CG30000, and CG30005). The G-site is shaded in blue. The α-helices and β-strands are indicated in red and green and labeled with α and β.

Total RNA was processed using RT-PCR. First-strand cDNA was produced using SuperScript II Reverse Transcriptase (Life Technologies, Carlsbad, CA) and an oligo-dT primer. The resulting cDNA was used as a PCR template with the oligonucleotide primers 5′-TATACCATGGTTTTAAAACTATATTATGAT-3′ (sense) and 5′-CCGGATCCTTAAAGTTTAGAATTAGCCGCA-3′ (antisense), based on a sequence obtained from the SilkBase EST database [20]. Underlined and double-underlined regions in the primer sequences represent *Nco*I and *Bam*HI restriction enzyme sites, respectively, which were used to insert the PCR product into an expression plasmid. PCR was performed with 1 cycle at 94°C for 2 min; then 35 cycles at 94°C for 1 min, 50°C for 1 min, and 72°C for 2 min; followed by 1 cycle at 72°C for 10 min. The resulting bmGSTT cDNA (*bmgstt*) was ligated into the pGEM-T Easy Vector (Promega, Madison, WI), which was then used to transform *E. coli* DH5α cells. Genetyx software (ver. 14.0.12, Genetyx Corp., Tokyo, Japan) was used to obtain the complete sequence of *bmgstt* and to deduce its corresponding amino acid sequence. Homology alignment (Fig. 1) was performed using ClustalW (ver. 1.83), with 10 and 0.2 as the gap creation penalty and gap extension, respectively. A phylogenetic tree was generated using neighbor-joining plot software (http://www-igbmc.u-strasbg.fr/Bioinfo/ClustulX/Top.html).

### Overexpression and purification of recombinant protein

The *bmgstt* clone was digested with *Nco*I and *Bam*HI and subcloned into the expression vector pET-11b, which was then used to transform competent *E. coli* Rosetta (DE3) pLysS cells (Novagen, EMD Biosciences, Inc., Darmstadt, Germany). Cells were then cultured at 37°C in Luria-Bertani media containing 100 µg/mL ampicillin. After cell density reached an OD_600_ of 0.7, isopropyl-1-thio-β-D-galactoside was added at a final concentration of 1 mM to induce recombinant protein production. The culture was further incubated for 3 h, and cells were harvested by centrifugation. Bacteria were resuspended in 20 mM Tris-HCl buffer (pH 8.0) containing 0.5 M NaCl, 4 mg/mL lysozyme, and 1 mM phenylmethanesulfonyl fluoride, and cells were subsequently disrupted by sonication. Unless otherwise stated, all operations for purification described below were conducted at 4°C. The supernatant containing the recombinant protein was clarified by centrifugation at 10,000×*g* for 15 min and subjected to ammonium sulfate fractionation. The pellet obtained by ammonium sulfate fractionation was resuspended in 20 mM Tris-HCl buffer, pH 8.5. After dialysis against the same buffer, samples were subjected to anion-exchange chromatography on a DEAE-Sepharose column (GE Healthcare Bio-Sciences AB, Uppsala, Sweden) and eluted with a linear gradient of 0–0.3 M NaCl. The enzyme-containing fractions, assayed as described below, were pooled, concentrated using a centrifugal filter (Millipore Corp., Billerica, MA), and applied to a Superdex 200 column (GE Healthcare Bio-Sciences, Buckinghamshire, UK) equilibrated with the same buffer, but with the addition of 0.2 M NaCl. The purity of the pooled material was analyzed by SDS-PAGE using a 15% polyacrylamide slab gel containing 0.1% SDS, according to the method of Laemmli [21]. Protein bands were visualized by Coomassie Brilliant Blue R250 staining, and protein concentrations were measured using a Protein Assay Kit (Bio-Rad Laboratories, Inc., Hercules, CA), with bovine serum albumin as a standard.

### Molecular modeling

A structural model of bmGSTT was constructed by SWISS-MODEL (http://swissmodel.expasy.org) [22] using the amino acid sequence. The model showed a GMQE (Global Model Quality Estimation) score of 0.69 [23]. The construction of the bmGSTT model was based on the structure of hGSTT1-1 (PDB code: 2C3T). The secondary structure assignments were produced with DSSP [24]. The Superpose program [25] revealed structural homology between bmGSTT and hGSTT1-1 with a root-mean-square deviation of 2.412 Å/214 residues for all atoms. Figures were prepared using Coot [26] and PyMOL (http://pymol.sourceforge.net).

### Site-directed mutagenesis

Amino acid-substituted mutants of bmGSTT were constructed using the Quick-Change Site-Directed Mutagenesis Kit (Stratagene Corp., La Jolla, CA), according to the manufacturer's recommendations. An expression plasmid containing *bmgstt* was used as a template, and full-length mutated cDNAs were verified by DNA sequencing.

### Measurements of enzyme activity

GST activity was spectrophotometrically measured using 1-chloro-2,4-dinitrobenzene (CDNB) and 5 mM GSH as standard substrates [27]. Enzymatic activity was expressed as mol CDNB conjugated with GSH per min per mg of protein. Alternatively, other substrates listed in Table 1 were used instead of CDNB [28], [29]. Kinetic parameters (*K*_m_ and *k*_cat_) were assessed with a nonlinear least-squares data fit under assay conditions with different substrate concentrations in the presence of 5 mM GSH. The kinetic parameters toward GSH were measured in the presence of 1 mM CDNB. Thermostability of bmGSTT was determined by pre-incubation of enzyme solutions at various temperatures for 30 min before a residual activity assay. The pH stability of bmGSTT was assessed by pre-incubation of enzyme solutions at various pH values at 4°C for 24 h before a residual activity assay. Optimal pH for bmGSTT activity was determined using citrate-phosphate-borate buffer at various pH values with a fixed ionic strength of 0.25.

**Table 1:** Substrate specificity of bmGSTT.

Substrate	Concentration (mM)	Activity (μmol/min/mg)	Wavelength (nm)	Δε (mM^−1^cm^−1^)
CDNB	1.0	0.03	340	9.6
EPNP	1.0	2.57	260	0.5
4NBC	1.0	NA	310	1.9
4NPB	1.0	3.56	310	1.2
4HNE	0.1	NA	224	13.8
ECA	1.0	NA	270	5.0
4NPA	1.0	NA	400	8.3
H_2_O_2_	0.2	NA	340	−6.2
PM	0.25	NA	---	---
DDT	0.1	NA	---	---
CP	0.25	NA	---	---

Activity was measured at pH 8 in the presence of 5 mM GSH. Data are expressed as means of three independent experiments. NA represents no activity. Wavelength and Δε represent maximum wavelength of the absorption and molecular coefficient, respectively. ---: not applicable.

### Insecticide metabolism assay

The ability of bmGSTT to metabolize each insecticide was determined by high performance liquid chromatography (HPLC) [14]. Reaction mixtures (500 µL) contained 120 µM PM, bmGSTT (12 µg), and 5 mM GSH in 50 mM Tris-HCl buffer at pH 8.0. Dehydrochlorinase activity for 1,1,1-trichloro-2,2-bis(4-chlorophenyl)ethane (DDT) was assayed by incubating the purified bmGSTT with 0.1 mM DDT and 5 mM GSH in 20 mM Tris buffer (pH 8.0) at 30°C for 2 h. DDT and its metabolites were analyzed by HPLC, as described below, according to a previous report [14], [30].

Reaction mixtures were extracted with three 500 µL portions of ethyl acetate for analysis by HPLC. After removing ethyl acetate, the amounts of each insecticide were determined by HPLC. An HPLC instrument (Prominence, Shimadzu Corp., Kyoto, Japan) was fitted with a 250×4.6 mm Mightysil RP-18 column (Kanto Chemical Co., Inc., Tokyo, Japan) with a flow rate of 1.0 mL/min at 40°C. The mobile phases employed were methanol (MeOH)/acetonitrile/H_2_O (70/15/15, v/v/v), MeOH/0.1% acetic acid (70/30, v/v), and MeOH/0.1% acetic acid (85/15, v/v) for detection of DDT, chlorfenapyr (CP), and permethrin (PM), respectively. The concentrations of each insecticide were determined from the corresponding peak areas identified by its authentic sample.

## Results

### Sequence of cDNA encoding bmGSTT

**Figure 2:**
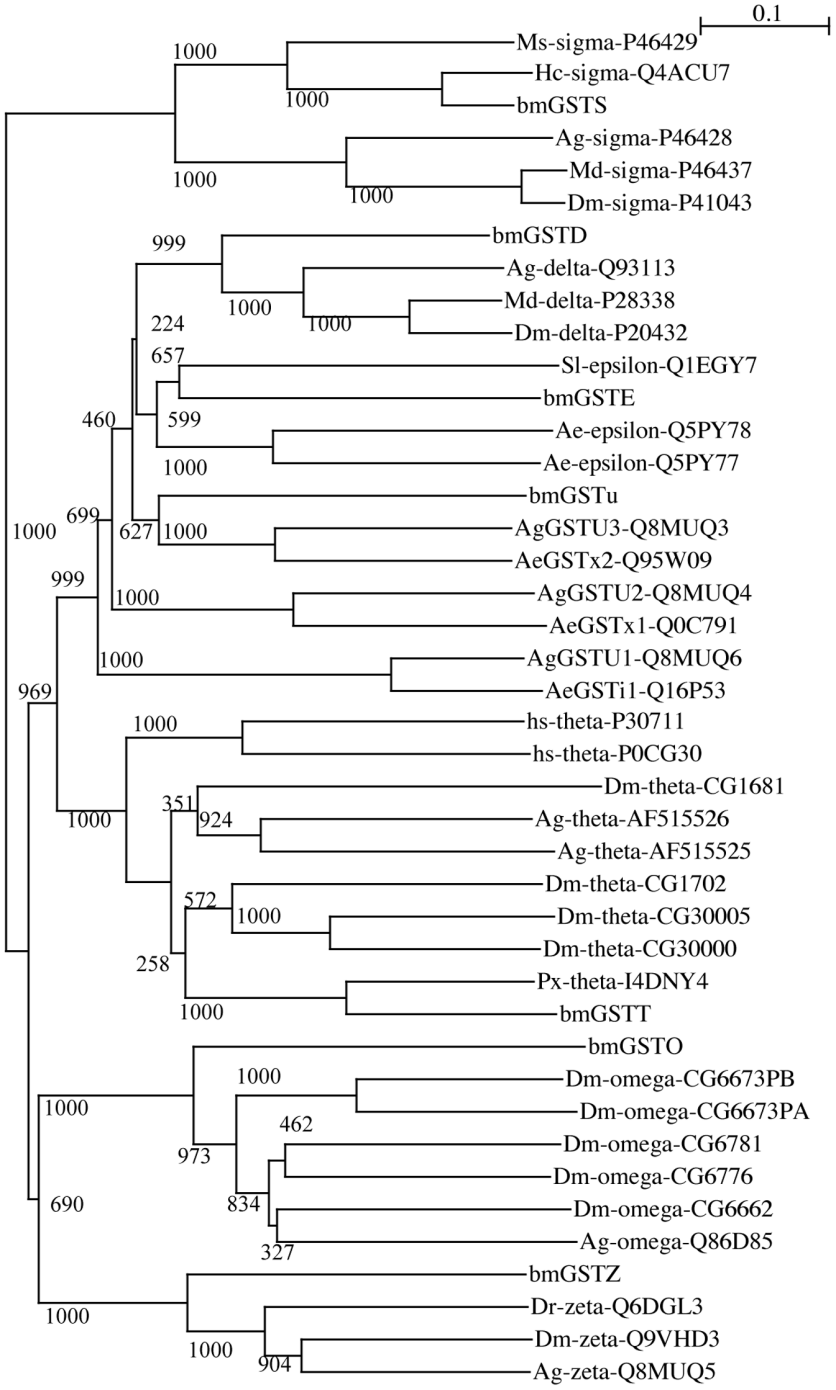
Phylogenetic analysis of amino acid sequences of GSTs. The phylogenetic tree was produced by neighbor-joining plot software using GST sequences obtained from NCBI (http://www.ncbi.nlm.nih.gov/), Swiss-Prot (http://expasy.org/sprot/), and flybase (http://flybase.org/); each entry contains the species name, GST class, and accession number. Numbers represent branch length; Ag, *A. gambiae*; Md, *Musca domestica*, Dm, *D. melanogaster*; Ms, *Manduca sexta*; Hc, *Hyphantria cunea*; Hs, *Homo sapiens*; bm, *B. mori*; Ae, *Aedes aegypti*; Sl, *Spodoptera litura*; Px, *Papilio xuthus*; and numbers attached to nodes indicate bootstrap values. The unclassified GST group does not include GST class.

We isolated a cDNA, *bmgstt*, from silkworm fat bodies, the nucleotide sequence of which is deposited in GenBank under Accession No. AB848737. A BLAST search (http://blast.ncbi.nlm.nih.gov/Blast.cgi) using the Swiss-Prot database (http://expasy.org/sprot/) showed that the sequence corresponds to theta-class GSTs. The sequence contains an open reading frame of 690 base pairs, encoding 229 amino acid residues (Fig. 1), and the deduced amino acid sequence shows 34% and 31% identities to hGSTT1-1 and hGSTT2-2, respectively. In *A. gambiae*, there are two isoforms of the theta class (GenBank under accession numbers: AF515525 and AF515526), whereas, in *D. melanogaster*, 4 isoforms of the theta class are known (flybase (http://flybase.org) under accession numbers: CG1681, CG1702, CG30000, and CG30005). The amino acid sequence of bmGSTT reveals identities of 39%, 45%, 30%, 47%, 35%, and 35% to AF15525, AF15526, CG1681, CG1702, CG30000, and CG30005, respectively (Fig. 1). Secondary structural elements of bmGSTT were predicted using SWISS-MODEL (http://swissmodel.expasy.org/workspace/). Additionally, the DSSP program [24] revealed that the bmGSTT monomer includes 9 α-helices and 4 β-strands (Fig. 1). In hGSTTs, there are additional α-helices, in comparison to bmGSTT, which would be present as α2a, α4c, and α9. The locations for the other α-helices and β-strands are conserved among the three GSTTs (Fig. 1). Construction of a phylogenetic tree clustered bmGSTT in the same clade as all current theta members (Fig. 2). The theoretical molecular mass and isoelectric point (26,913 Da and 8.91, respectively) of bmGSTT are similar to those of zeta- and delta-class GSTs from *B. mori* (Table 2).

**Table 2:** Properties of GSTs as determined in the present and previous studies.

class	theta	epsilon	unclassified	delta	omega	sigma	zeta
Calculated Molecular Weight (Da)	26,913	25,296	24,457	24,225	29,806	23,338	24,727
Calculated Isoelectric Point	8.91	5.98	7.80	8.35	6.01	5.79	8.5
Optimum pH	8	8	4–9	8	7–8	8	6
Stable pH Range	5–11	5–10	5–9	3–9	2–12	4–11	4–10
Stable Temperature Range	<50°C	<50°C	<50°C	<50°C	<50°C	<40°C	<50°C
Substrate Specificity	CDNB EPNP 4NPB	CDNB ECA GPx	CDNB	CDNB 4HNE 4NPA	CDNB 4HNE GPx	CDNB	DCA

Delta, *B. mori* delta-class GST; epsilon, *B. mori* epsilon-class GST; omega, *B. mori* omega-class GST; sigma, *B. mori* sigma-class GST; unclassified, *B. mori* unclassified GST; zeta, *B. mori* zeta-class GST. Data were obtained from previous reports ([13] for delta; [14] for epsilon; [15] for omega; [16] for sigma; [18] for unclassified; [17] for zeta). DCA, dichloroacetic acid.

### Putative GSH-binding site (G-site)

**Figure 3:**
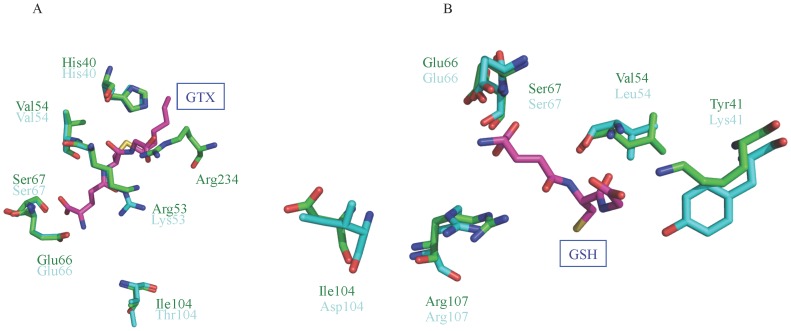
Superimposed structures of bmGSTT with hGSTT1-1 (A) and hGSTT2-2 (B) showing amino acid residues of the G-site. Carbon atoms for bmGSTT, hGSTTs, and GTX/GSH are green, cyan, and magenta, respectively, except for the regions of oxygen (red), nitrogen (blue), and sulfur (yellow). Symbols of amino acid residues for bmGSTT and hGSTTs are shown in green and cyan, respectively.

The G-site identified in hGSTT1-1 include His40, Val54, Lys53, Glu66, Ser67, and Thr104; whereas in hGSTT2-2, they are Lys41, Leu54, Glu66, Ser67, Asp104, and Arg107 [31]. Superimposition of modeled bmGSTT on hGSTT1-1 indicates that equivalent *B. mori* residues include His40, Arg53, Val54, Glu55, Ser67, and Ile104 (Fig. 3A). In this model, the distance is large between the oxygen atom in the cysteinyl region of *S*-hexylglutathione (GTX), an inhibitor of GST, and the side-chain of Arg53 of bmGSTT (3.54 Å), and there is a large distance between the amino group in the γ-glutamyl region of GTX and the side-chain of Ile104 (8.79 Å) of bmGSTT. In hGSTT2-2, GSH interacts with amino acid residues (Lys41, Leu54, Glu66, Ser67, Asp104, and Arg107) (Fig. 3B), which are superimposed upon Tyr41, Val54, Glu66, Ser67, Ile104, and Arg107 in bmGSTT. In Fig. 3B, GSH is far away from Tyr41 (3.41 Å between oxygen atom in the glycine portion of GSH and the hydroxy-group of Tyr41) and Ile104 (8.39 Å between the amino-group in the γ-glutamyl region of GSH and the side-chain of Ile104) in bmGSTT. Taken together, the structural data indicate that five residues (His40, Val54, Glu66, Ser67, and Arg107) in bmGSTT participate in the interaction with GSH.

### Characterization of bmGSTT

**Figure 4:**
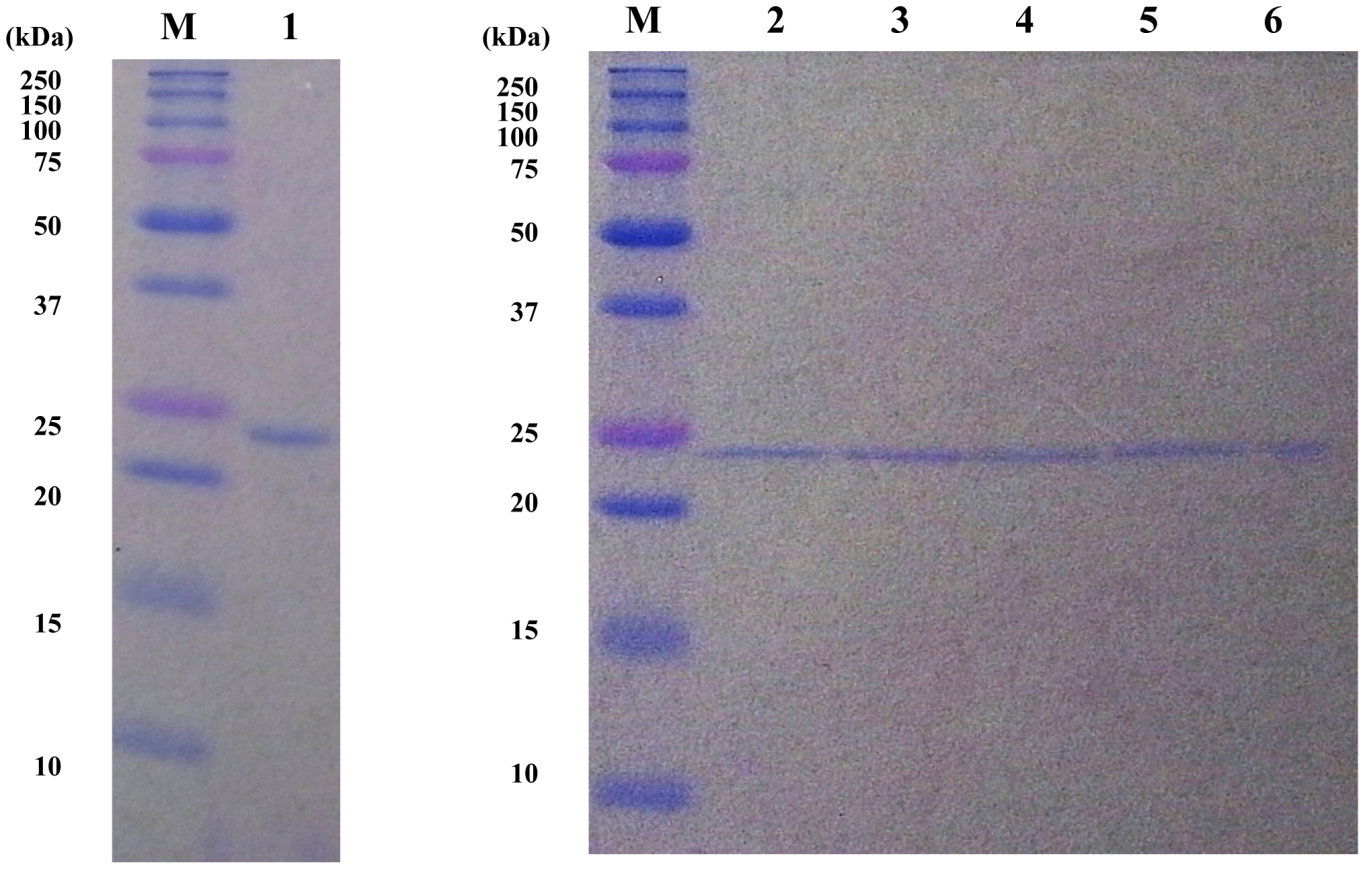
Recombinant bmGSTT and its mutants overexpressed in *E. coli*. bmGSTT mutants were prepared by site-directed mutagenesis. Purified recombinant proteins were applied to SDS-PAGE followed by Coomassie Brilliant Blue R250 staining; M, protein molecular size markers; lane 1, WT; lane 2, H40A; lane 3, E66A; lane 4, S67A; lane 5, R107A; and lane 6, V54A.

Bacterially produced bmGSTT was purified to homogeneity, yielding a single band in SDS-PAGE with a molecular size of approximately 26,000 Da (Fig. 4). This size is close to the estimated size based on the deduced amino acid sequence.

The enzymatic properties of purified bmGSTT were studied using CDNB and GSH as substrates. The enzyme was stable at temperatures <50°C and retained >75% of its original activity over a pH range of 5–11, similar to other *B. mori* GSTs. The pH optimum of bmGSTT was 8.0, which is identical to the optima of epsilon-, delta-, and sigma-class *B. mori* GSTs.

Various substrates were then used to profile the activity of bmGSTT. Table 1 shows that bmGSTT was active toward CDNB, 1,2-epoxy-3-(4-nitrophenoxy)-propane (EPNP), and 4-nitrophenethyl bromide (4NPB), but it was not active toward 4-nitrobenzyl chloride (4NBC), 4-hydroxynonenal (4HNE), ethacrynic acid (ECA), or 4-nitrophenyl acetate (4NPA). The activity of bmGSTT toward CDNB is higher than that of GSTT-1 from rats (0.01 µmol/min/mg), mice (0.01 µmol/min/mg), or humans (0.005 µmol/min/mg) [32]. The activity of bmGSTT toward EPNP and 4NPB is lower than those of rat and mouse GSTTs but higher than that of hGSTT1-1[32]. 4HNE is a preferred substrate for delta bmGST and omega bmGST (bmGSTO) (Table 2), whereas bmGSTT was relatively inactive against 4HNE. GSH peroxidase (GPx) activity was not observed in bmGSTT, unlike that in bmGSTO and epsilon bmGST. Subsequent HPLC analysis revealed that bmGSTT was unable to recognize DDT, CP, and PM as substrates (Table 1).

### Amino acid residues involved in catalytic function

Based on the G-site of hGSTT1-1 and hGSTT2-2, we identified His40, Val54, Glu66, Ser67, and Arg107, as the candidate G-site of bmGSTT (Figs. 1 and 3). To determine whether these residues are important for catalytic activity, we performed site-directed mutagenesis. The resulting mutants were named H40A, V54A, E66A, S67A, and R107A and were purified from *E. coli* clones (Fig. 4). Each preparation of mutant enzyme was present as a single band in SDS-PAGE. Since the activity of bmGSTT toward EPNP and 4NBC did not fit the Michaelis-Menten equation, we determined kinetic parameters with CDNB, GSH, and 4NPB and compared these parameters with those of the wild-type (WT) enzyme (Table 3). With CDNB as the substrate, the enzyme's *K*_m_ was 1.5 mM, which was 3.8-, 3.1-, 2.2-, and 0.96-fold the value for unclassified, delta-, omega-, and sigma-class GSTs, respectively [13], [15], [16], [18]. The *K*_m_ values for V54A, E66A, and S67A were higher than that of WT. The *k_cat_* values for V54A, E66A, and S67A were higher, while the values for other mutants were lower, compared to that of WT. The *k_cat_/K*_m_ values from E66A and S67A were 41% and 28% of that of WT, respectively, and no large differences in *k_cat_/K*_m_ values were observed for H40A, V54A, and R107A. With GSH as the substrate, the *K*_m_ values for V54A and E66A were 3.1 and 3.6 times that of WT, whereas no *K*_m_ could be calculated for the S67A mutant. The *k_cat_/K*_m_ value of S67A was undetectable, whereas that for E66A decreased by 54%; no marked changes in *k_cat_/K*_m_ values were observed for H40A, V54A, and R107A. With 4NPB as the substrate, the *k_cat_/K*_m_ values for H40A and R107A were 22% and 40% of that of WT, respectively; a similar value was observed for V54A. For E66A and S67A, we were unable to detect the *kcat/K*_m_ value with 4NPB. In summary, the most distinctive features of this mutagenesis are the decreased *k_cat_/K*_m_ values toward CDNB, GSH, and 4NPB for S67A, compared to those of WT. These results suggest that the interaction between GSH and Ser67 of bmGSTT is crucial for the activity.

**Table 3:** Comparison of kinetic data from bmGSTT and mutant forms.

		bmGSTT mutants				
	WT	H40A	V54A	E66A	S67A	R107A
CDNB						
*K*_m_ a	1.5 (0.25)	0.52 (0.080)	28 (3.6)	12 (2.1)	4.3 (0.72)	0.96 (0.11)
*k_cat_* ^ b^	0.27 (0.020)	0.12 (0.036)	3.9 (0.84)	0.88 (0.85)	0.35 (0.022)	0.16 (0.042)
*k_cat_/K*_m_^c^	0.18	0.23	0.22	0.073	0.051	0.17
GSH						
*K*_m_ a	3.6 (0.56)	1.8 (0.34)	11 (1.9)	12.9 (2.2)	ND	3.9 (0.35)
*k_cat_* ^ b^	0.15 (0.034)	0.065 (0.018)	0.52 (0.082)	0.28 (0.077)	ND	0.13 (0.022)
*k_cat_/K*_m_^c^	0.041	0.036	0.049	0.022	ND	0.033
4NPB						
*K*_m_ a	0.13 (0.03)	2.3 (0.22)	0.50 (0.083)	ND	ND	0.52 (0.071)
*k_cat_* ^ b^	0.78 (0.17)	3.1 (0.56)	3.7 (0.61)	ND	ND	1.3 (0.16)
*k_cat_/K*_m_^c^	6.0	1.3	7.4	ND	ND	2.4

Values, except those of *k_cat_/K*_m_, are expressed as means of three independent experiments.

a Expressed in units of mM; ^b^expressed in units of μmol/L/min; and ^c^expressed in units of/min/mM. ND represents ‘not detected’.

## Discussion

Although many GSTs have been identified in *B. mori*, the theta class remains poorly understood. This is a critical gap in our knowledge, because understanding the metabolic profile of theta-class GSTs may provide novel insecticide-targeting strategies. According to the silkworm genome sequence, there could be 23 homologs of GSTs: delta-class (4 isoforms), epsilon-class (8 isoforms), omega-class (4 isoforms), sigma-class (2 isoforms), theta-class (1 isoform), zeta-class (2 isoforms), and unclassified (2 isoforms) GSTs. In the *A. gambiae* genome, the GST classes include delta-class (12 isoforms), epsilon-class (8 isoforms), omega-class (1 isoform), sigma-class (1 isoform), theta-class (2 isoforms), zeta-class (1 isoform), and unclassified (3 isoforms) GSTs, whereas, in *D. melanogaster*, the classes include delta-class (11 isoforms), epsilon-class (14 isoforms), omega-class (5 isoforms), sigma-class (1 isoform), theta-class (4 isoforms), zeta-class (2 isoforms), and no unclassified (0 isoform) GSTs. The silkworm genome contains a single gene encoding a theta-class GST. Previously, we reported identification of one theta-class GST of *B. mori* [13], which has been recently reassigned to the delta class. Thus, the focus of this study was on a silkworm GST in the theta class, which had not been thoroughly investigated, in terms of molecular and biochemical properties.

GSTs catalyze a broad range of reactions, and each family member has its own discrete substrate specificity. This characteristic is also true for *B. mori* GSTs (Table 2). bmGSTT possesses GSH-conjugation activities toward EPNP and 4NPB, a property shared with mammalian theta-class GSTs. In contrast to hGSTT1-1, bmGSTT was not reactive with 4NBC and H_2_O_2_, suggesting that the catalytic properties of the bmGSTT enzyme are unique. bmGSTT did not recognize 4HNE, a cytosolic product of lipid peroxidation [33], or H_2_O_2_ as substrates, indicating that the enzyme is unlikely to participate in the response to oxidative stress. Intriguingly, although bmGSTT shares some substrate preferences with mammalian GSTTs, it appears to have very different substrate specificity compared to other *B. mori* GSTs. Epsilon-class GSTs of mosquito could be involved in resistance to DDT and pyrethroid insecticides [34], [35]. This resistance is particularly relevant given that HPLC analyses revealed that bmGSTT was unable to degrade the insecticides tested, in contrast to the results with other *B. mori* GSTs.

The GST amino acid sequence is divided into two regions, the N- and C-terminal domains [5]. The N-terminal domain includes the G-site, and the C-terminal domain has a hydrophobic substrate-binding site (H-site). The sequence diversity of the H-site dictates substrate selectivity [5]; moreover, this diversity likely explains the varied substrate specificity of *B. mori* GSTs, because there is considerable divergence between their C-terminal regions (alignments not shown). Our mutagenesis results suggest that residues Glu66 and Ser67 in bmGSTT play important roles in its catalytic functions. Notably, while mutation of His40 in bmGSTT did not alter the kinetics of catalysis, the equivalent residue in delta- and epsilon-class GSTs is critical for GSH binding [14], [36]. The mutation to Val54 had a minor effect on enzyme catalysis. This result was expected, because the mutation affected the main chain of the residue that interacts with GSH and not the side chain. We assume that His40 and Arg107 are not entirely crucial for binding of GSH and, instead, play co-operative roles with other residues in the G-site of bmGSTT. Similar observations were reported for an unclassified GST of *B. mori* (bmGSTu) [37], in which the equivalent residue (His53) of bmGSTu interacts with pre-bound GSH, but the mutation of the His to Ala did not affect catalytic activity.

As mentioned above, the diversity of amino acids at the N- and C-terminal binding domains of GST is associated with substrate selectivity. hGSTT1-1 contains an H-site formed by Leu7, Leu35, Ile36, His40, Leu111, Trp115, Met119, Phe123, His176, Leu231, Trp234, Val235, and Met238 [32]. We found that only 3 of these 13 residues were conserved in the H-site of bmGSTT, which may explain the difference in substrate specificity between bmGSTT and hGSTT1-1. Additionally, a C-terminal helix in theta-class GSTs and residue 234 in the amino acid sequence of hGSTT1-1 play important roles in substrate specificity and catalysis, respectively [31], [32] [38]. There is no corresponding region, including the residue at position 234, in bmGSTT (Fig. 1), which may explain why it exhibits lower activity than rat, mouse, and human theta-class GSTs [39].

Recently, the electron-sharing network that contributes to the catalytic activity of GST has been described [40], [41]. Based on an amino acid residue at position 64 that is functionally conserved in the GST classes [40], this network can be divided into type I and II classes. The type I electron-sharing network is exemplified by delta-, theta-, omega-, and tau-class GSTs, which contain an acidic amino acid residue at position 64, whereas the type II network GSTs (alpha, mu, and pi classes) have a polar amino acid residue. Glu66 is conserved in the sequence of bmGSTT; thus, this enzyme resembles a member of the type I network. The electron-sharing network in hGSTT2-2 was proposed to contain Ser67 as one of residues involved in the network [41]. The equivalent residue in bmGSTT (Ser67) is conserved (Fig. 1). Glu66 and Ser67 in bmGSTT could be part of an electron-sharing network and the G-site via direct interaction with GSH. Thus, mutation of the residues may result in a decrease in GSH-conjugation activity.

Other than five residues (His40, Val54, Glu66, Ser67, and Arg107) in bmGSTT, there could be other amino acid residues that are essential for bmGSTT catalytic activity. In theta-class GSTs, the Ser residue in the N-terminal domain is conserved [42]–[44] and considered important for activation of the bound GSH. The equivalent residue in bmGSTT is Ser11 (Fig. 1). In other GST classes, mutagenesis of amino acid residues in electron-sharing networks results in decreased activity [40], [41]. Investigation of putative catalytic residues using site-directed mutagenesis is now underway in our laboratories.

Our results suggest that bmGSTT might play a role in detoxification of xenobiotics in *B. mori*. Together with bmGSTT, the roles of other GSTs in *B. mori* should be further examined to understand the mechanisms underlying insecticide detoxification. In turn, such studies will aid the design and implementation of insecticide-resistance management strategies for agricultural pests.
